# Granulomatous and xanthogranulomatous prostatitis: A case report

**DOI:** 10.1016/j.eucr.2021.101887

**Published:** 2021-10-12

**Authors:** Al-Naimi A, M. Zaki Karzoun, Osama Abdelfattah, Tarek Ibrahim

**Affiliations:** a Department of Surgery, Urology Section, Hamad Medical Corporation, Doha, Qatar; b Department of Pathology, Hamad General Hospital, Hamad Medical Corporation, Doha, Qatar

**Keywords:** Granulomatous, Xanthogranulomatous, Prostatitis, TURP, Trans-Urethral Resection of the Prostate, XGP, Xanthogranulomatous prostatitis, LUTS, Lower Urinary Tract Symptoms, PSA, prostate-specific antigen, CT, computed tomography, MRI, magnetic resonance imaging, mpMRI, multiparametric MRI, BPH, benign prostatic hyperplasia, UTI, urinary tract infections

## Abstract

Granulomatous prostatitis is a rare condition that is diagnosed only by histopathological examination. Though rare, the condition was reported to have different presentations (mimicking prostate cancer or prostatitis and prostatic abscess) and to have different etiologies which classified it into three main entities; nonspecific (idiopathic), post-surgery, and specific. Specific granulomatous prostatitis is further sub-classified to infective, xanthogranulomatous, Malacoplakia and associated with systemic granulomatous disease and allergy. We hereby report a rare case of xanthogranulomatous prostatitis that presented with persistent urinary tract infection.

## 1 Introduction

Granulomatous prostatitis, which was first described in 1943,^1^ is rarely encountered in urologic practice. Nonspecific granulomatous prostatitis is the most common type of granulomatous lesion of the prostate, followed by tuberculous prostatitis and granulomatous lesions following transurethral resection of the prostate (TURP) then specific granulomatous lesions including Xanthogranulomatous prostatitis (XGP).^2^ XGP which is very rare, may present asymptomatically, or may mimic prostatic abscess or prostatic carcinoma making histological verification mandatory.^3^ This report describes a patient diagnosed as XGP with concomitant nonspecific granulomatous prostatitis.

## 2 Case presentation

A 63-year-old male patient with diabetes and hypertension presented with a 2-year history of lower urinary tract symptoms (LUTS). He was found to have an obstructed infected kidney secondary to a right upper ureteric stone, for which he underwent percutaneous drainage followed by retrograde intrarenal surgery. His LUTS, however, worsened, and he experienced recurrent attacks of epididymo-orchitis together with urine cultures confirming *Escherichia coli* (*E. coli*).

Upon presentation six months later, his general physical condition was normal. A digital rectal examination revealed asymmetrical enlargement of the left lobe of the prostate. His prostate-specific antigen (PSA) concentration was initially elevated (6 ng/ml) but normalized to 1.7 ng/ml after a course of antibiotics.

Ultrasound examination showed an enlarged prostate (29 cc) with a prominent median lobe. A computed tomography (CT) scan with contrast showed an enlarged prostate with prostatic calcification, and magnetic resonance imaging (MRI) of the pelvis showed benign prostatic hyperplasia (BPH) with evidence of changes due to chronic prostatitis. His urine flow rate was 6 ml/seconds and his post-void residual urine volume was 90 ml. Cystoscopy examination showed tri-lobar enlargement of the prostate with severe bladder wall trabeculation and the formation of multiple diverticula.

Based on these findings, including progressive LUTS, recurrent urinary tract infections (UTI), and low flow rate, TURP was performed. His intraoperative and postoperative course was uneventful, with a postoperative maximum urine flow rate of 23 ml/second.

**Fig. 1:**
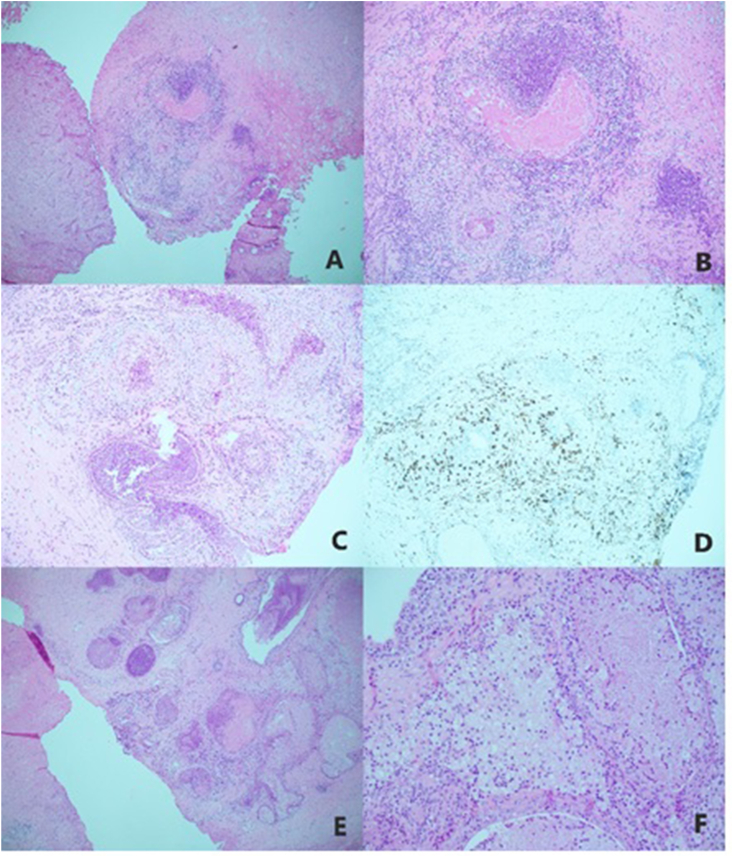
Histopathologic examination of tissue samples from this patient. (A,B) Proliferating epithelioid cells surrounded by lymphocytes forming poorly defined granulomas at (A) 4X and (B) 10X magnification of the same focus. (C) Granulomatous inflammation with neutrophil infiltration at 10X magnification. (D) Immunohistochemistry with anti-CD68 antibodies, showing staining of epithelioid cells. (E.F), Dense infiltration of foamy histiocytes, accompanied by lymphocytes, plasma cells, eosinophils, and neutrophils at (E) 4X and (F) 20X magnification of the same focus.

Histopathologic examination of the resected tissue revealed granulomatous and xanthogranulomatous prostatitis (Fig. 1). BPH was diagnosed, with no evidence of malignancy.

## 3 Discussion

Although granulomatous prostatitis was first described in 1943, the pathology of XGP was first reported in 1986.^1^ Several methods have been utilized to date to classify granulomatous lesions of the prostate, with these lesions currently classified as described previously.^2^

Our patient presented with persistent urinary tract infection and had suspicious findings on digital rectal examination of the prostate along with elevated PSA. XGP is a rare entity that cannot be differentiated clinically from other forms of chronic prostatitis and may mimic prostate cancer.^3^ A concomitant XGP may even coexist with a prostate abscess and prostate cancer in the same patient.^4^ Hence, XGP can be confirmed only by histopathological examination. It is treated mainly by supportive therapy, with TURP performed in patients with severe lower urinary tract obstruction. Follow-up should include measurements of PSA and, if necessary, needle biopsy of the prostate.^4^ In the present patient, PSA became normal after treatment with antibiotics.

Granulomatous prostatitis and poorly differentiated carcinoma can be reliably distinguished by immunohistochemical methods.^4^ XGP is distinguished from other, similar conditions by the presence of large numbers of “foamy macrophages “(histiocytes) that form granulomas. Other inflammatory cell infiltrates (Lymphocytes and plasma cells) may be present but are non-specific; present in other chronic inflammatory prostatic conditions.^4^

Despite difficulties distinguishing granulomatous prostatitis from adenocarcinoma, even on multiparametric MRI (mpMRI), XGP is suggested by evidence of diffuse changes involving >50% of the prostate gland associated with infiltration of periprostatic fat or extracapsular extension, the presence of large areas of nonenhancement corresponding to caseous abscesses and of areas of rim enhancement.^5^ MRI of the pelvis in this patient showed BPH with evidence of chronic prostatitis changes.

Histopathology is crucial in the diagnosis of XGP or to exclude the presence of carcinoma. Specimens can be obtained from transrectal ultrasound biopsy or after performing TURP. Histopathologic examination of this patient showed granulomatous and xanthogranulomatous prostatitis, but no evidence of concomitant prostate cancer.

## 4 Conclusion

XGP is a very rare disease that mimics prostate adenocarcinoma. Careful assessment is required to differentiate these two diseases. Abnormal results on digital rectal examination and elevated PSA indicate the need for further evaluations, such as MRI and transrectal biopsy, to exclude the presence of prostate cancer.

## Declaration of competing interest

There are no conflicts of interest.
